# Galectins as New Prognostic Markers and Potential Therapeutic Targets for Advanced Prostate Cancers

**DOI:** 10.1155/2013/519436

**Published:** 2013-09-24

**Authors:** Diego J. Laderach, Lucas Gentilini, Felipe M. Jaworski, Daniel Compagno

**Affiliations:** Laboratorio de Glicómica Funcional, IQUIBICEN-CONICET, Departamento de Química Biológica, Facultad de Ciencias Exactas y Naturales, Universidad de Buenos Aires, C1428, Buenos Aires, Argentina

## Abstract

A better understanding of multimolecular interactions involved in tumor dissemination is required to identify new effective therapies for advanced prostate cancer (PCa). Several groups investigated protein-glycan interactions as critical factors for crosstalk between prostate tumors and their microenvironment. This review both discusses whether the “galectin-signature” might serve as a reliable biomarker for the identification of patients with high risk of metastasis and assesses the galectin-glycan lattices as potential novel targets for anticancer therapies. The ultimate goal of this review is to convey how basic findings related to galectins could be in turn translated into clinical settings for patients with advanced PCa.

## 1. Introduction

Prostate cancer (PCa) is the second most common cancer in men and represents a significant cause of mortality worldwide [1]. About 15%–20% of men with PCa will certainly develop metastatic disease and die. Early diagnosis and rapid treatment play a critical role in the final outcome of the disease. At present, surgical and radiation treatments are efficient against clinically localized PCa, whereas androgen ablation is mainly recommended for advanced PCa [2]. However, metastatic cancer is essentially fatal due to disease evolution towards a castration-resistant PCa (CRPC). Novel alternative approaches are therefore essential to prevent tumor dissemination and progression to this incurable stage. 

Effective cancer therapies for PCa typically capitalize on molecular differences between healthy and neoplastic tissues that can be targeted with drugs [3]. In the past years, delineating gene and protein expression profiles has been critical in dissecting the molecular underpinnings of cellular function; the arising information has been exploited for the design of rational therapeutic strategies. In the postgenomic era, the study of the “glycome” has enabled the association of specific glycan structures with the transition from normal to neoplastic tissue [4]. Glycans abundantly decorate the surface of all mammalian cells and the extracellular matrix with which they interact [5]. In general, mammalian glycans are the product of a repertoire of glycosyltransferases and glycosidases acting sequentially and dictating the glycosylation signature of each cell type [6]. It has been recognized that the structure of cell surface glycans can change under different physiological and pathological conditions. In fact, malignant transformation is associated with abnormal glycosylation resulting in the synthesis of altered glycan determinants in the tumor microenvironment [7]. The responsibility of decoding the information displayed by cell surface glycan structures is attributed in part to endogenous glycan-binding proteins or lectins, whose expression and function are also regulated during oncogenesis and metastasis [8].

Galectins (Gals) are a family of evolutionarily conserved glycan-binding proteins characterized by their affinity for N-acetyllactosamine sequences which can be displayed on cell surface glycoconjugates [9, 10]. Through this type of interactions, Gals promote lattice formation, strengthening the avidity and half-life of ligand/receptor interactions, and organize centers for molecular signaling [11]. Therefore, these particular lectins are the molecular links between changes in glycophenotype and signaling processes that underlie cellular responses to exogenous stimuli. In addition, Gals are also involved in endogenous regulation of different intracellular pathways with high impact on controlling cellular behavior [12–14]. 

Interestingly, alterations in Gal expression are observed in pathologic processes such as inflammation, cancer, and autoimmunity [9, 15–17]. A series of studies in experimental models and cancer patients have reported significant associations among the expression of Gals and tumorigenesis, metastatic potential, and tumor-immune escape. This review focuses on the role of Gals in PCa progression and how could they be used as diagnostic markers of PCa evolution as well as new therapeutic targets for metastatic and castration-resistant PCa (mCRPC) patients.

## 2. Basic Biochemistry and Molecular Biology of Galectins

Galectins are a family of fifteen described lectins that bind to the carbohydrate portion of cell surface glycoproteins or glycolipids and are defined by at least one carbohydrate recognition domain (CRD) with affinity for beta-galactosides through a conserved sequence motif [18]. However, each of the members of this family has subtle differences in their glycan-binding specificity and tissue distribution (for detailed information see The Center of Functional Glycomics (CFG) database http://www.functionalglycomics.org/CFGparadigms/index.php/Main_Page). Members of the Gal family are found in vertebrates, invertebrates, and protists; Gal-related sequences have also been found in plants and viruses. The high degree of conservation of Gal sequences suggests that they have an important role in basic cellular mechanisms [19, 20]. However, from the fifteen defined members only 11 are expressed in humans (see Table 1, [18]).

**Figure 1:**
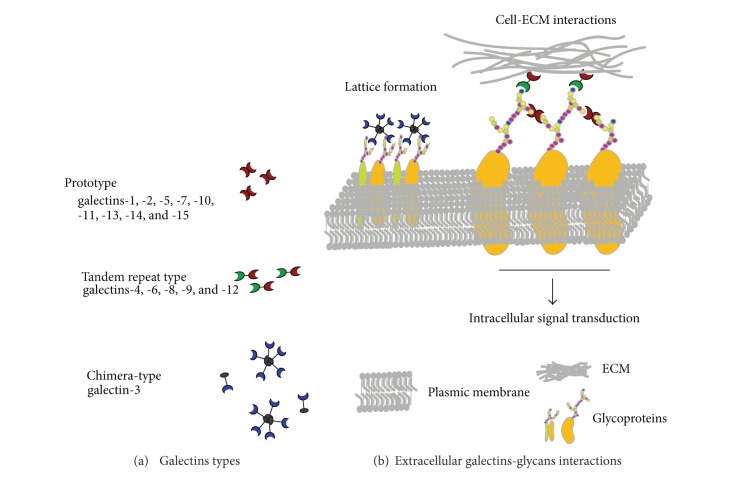
Interactions of galectins with extracellular glycoconjugates.

**Figure 2:**
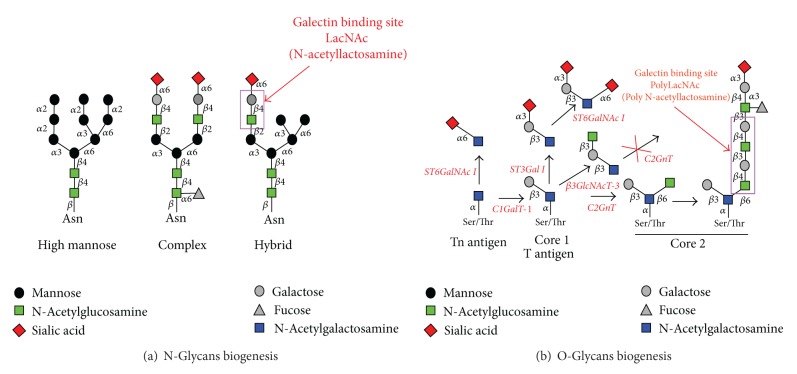
Glycans biogenesis and galectins recognition. (a) N-Glycans and (b) O-Glycans.

Galectins have been subdivided into three groups (Figure 1(a)) based on their structure and the number of CRD: (a) prototype Gals are constituted by a single CRD (Gals-1, -2, -5, -7, -10, -11, -13, and -14); (b) tandem-repeat Gals, by 2 different but homologous CRDs, connected by a linker region (Gals-4, -6, -8, -9, and -12); (c) chimera-Gals, represented by a unique member: Gal-3, consisted by a single CRD fused to a tail of short tandem repeats. These different structures allow oligomerization of Gals required for effective signaling through binding to cell surface glycoconjugates containing N-acetyllactosamine moieties (Figures 1(b) and 2) [11]. As an example, Gal-3 is a chimera-type Gal containing a single carbohydrate recognition domain (CRD) with an extended proline and glycine rich-N-terminus that promotes oligomerization towards highly structured forms. As other Gals, extracellular and membrane triggered functions of Gal-3 strongly depend on CDR-mediated recognition of glycan chains on glycoproteins, inducing the assembly of lattices in membrane through direct engagement of specific cell surface glycoconjugates by traditional ligand-receptor binding [10].

Galectins are synthesized in the cytoplasm and secreted using a nonclassical pathway [44]; thus, these lectines are found in a variety of intracellular compartments, as well as in the extracellular milieu of almost all cell types and tissues. Membrane-bound or soluble forms of Gals have been described with some different functions: it is well established that glycoconjugate recognition by Gals plays key roles during anoikis resistance, metastatic dissemination, and escape of tumor cells from the immune response (see for review [9, 45]). 

Galectins could be affected by posttranscriptional modifications such as cleavage or phosphorylation. In fact, phosphorylation or proteolysis affects Gal-3 structure and localization altering important biologic functions of this lectin in human carcinomas [46–48]. Gal-3 cleavage by matrix metalloprotease (MMP)-2/-9 is observed in breast and prostate cancers and is responsible for tumor growth, angiogenesis, and apoptosis resistance in mouse models and influence cell migration, angiogenesis, and tumor progression [48, 49]. 

The expression pattern of different Gals changes during tissue development and is altered at sites of inflammation and tumor. Different reports in colon, breast, prostate, thyroid, and laryngeal cancers have demonstrated an important role of Gals in tumor emergence and progression (see for review [15]). At this respect, the expression level of some Gals by tumor cells has been shown to be correlated with metastatic potential. Gals can contribute to tumor progression through many different mechanisms [50, 51]. Most studies have evaluated defined Gals, particularly Gals-1 and -3. However, existing data indicate that other Gals, especially those whose expression is altered in cancer, probably contribute to various steps in tumor progression. Mechanisms by which those Gals are involved in such effects remain poorly understood especially in the PCa field.

## 3. Expression of Galectins in Normal and Cancerous Human Prostate Tissues 

**Figure 3:**
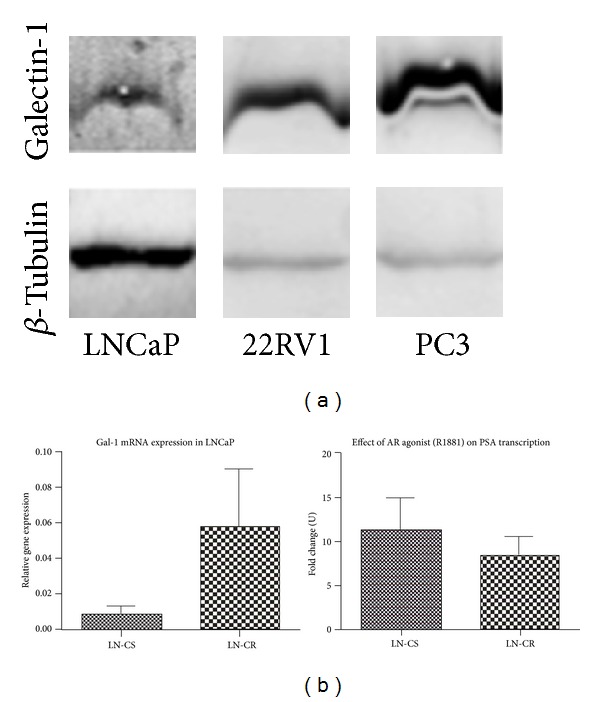
Galectin-1 expression in LNCaP cells. (a) Protein levels of Gal-1 in castration sensitive LNCaP and castration resistant 22Rv1 and PC-3 PCa cell lines. (b) Transcriptional levels of Gal-1 in castration sensitive (CS) or resistant (CR) LNCaP cells. Induction of prostate specific antigen (PSA) in response to androgen receptor agonist (R18.81; 3 days, 10^−10^ M) is shown in both cases as fold change between cultures in absence of hormones and in presence of R18.81. Cells were cultured in absence of hormones (medium complemented with 10% stripped charcoal-treated serum) for 48 h and then cultured for 3 days in absence or presence of R18.81 (10^−10^ M) before mRNA extraction and RT-qPCR for Gal-1 and PSA expression analyses.

Few studies investigated the expression of Gals in normal prostate [52, 53] and Gals-1; -3, and -8 are the most studied proteins in prostate carcinoma [23, 29, 52–55]. Pioneer studies by Lotan's group found that Gal-1 and Gal-3 are expressed in the cytoplasm of the majority of prostate cancer cell lines, except LNCaP which is the most studied human androgen-sensitive cell line. In fact, LNCaP does not express both Gals [25]. It is important to note that although LNCaP cells were clearly negative for Gal-1 expression in this first published work, we and others have demonstrated that LNCaP cell line does express low levels of Gal-1 mRNA and these transcripts are upregulated when cells underwent castration resistance (CR) [56]. In our study, we were able to detect the expression of Gal-1 at transcriptional levels with a 20-fold lower level when compared with androgen unresponsive 22Rv1 and PC-3 cells (Figure 3(a)). Additionally, we have been able to detect low protein levels by immunocytochemistry and western blot (see Figure 3(a)). Even more, LNCaP cells that have been rendered androgen unresponsive (by culturing them for several weeks in the absence of hormones) expressed higher levels of Gal-1 as detected by RT-qPCR (see Figure 3(b), left panel). Our studies have been performed on cells in the log phase of growth, as we used to observe variation of Gal expression depending on cell culture conditions such as confluency (data not shown), situation that is not referred to in previous cited references. Moreover, two different sources of androgen-responsive and PSA/Gal-1-producing LNCaP cells were evaluated as we do infer that some phenotypic differences may appear in such largely used PCa cell line (Figure 3(b), right panel). Results clearly demonstrate that the LNCaP cell line (either castration-sensitive or a clone that undergoes castration-resistant) as well as a PSA-negative LNCaP clone (selected *in vitro* by Vaarala and colleagues [56]) is able to produce Gal-1, at least under some culture conditions. However, when expressed, Gal-1 plays an important function in cell-extracellular matrix (ECM) interactions, conferring adhesion properties to some PCa cell lines. Gals-1 and -3 have been thus suggested to play a role in the development and progression of cancer [25].

Studying the role of Gals in PCa cell lines is certainly not enough to validate relevance in the human pathology. For clinical relevance, Lotan's group has studied by immunohistochemistry (IHC) the expression of Gals in normal human prostate tissue and prostate adenocarcinoma, containing formalin-fixed, paraffin-embedded sections of 7 normal human prostates, 8 cases of prostatic intraepithelial neoplasia (PIN), 20 primary adenocarcinomas of the prostate, and 12 PCa metastases. Gal-1 was expressed in most cases, irrespective of the histology stages. In contrast, Gal-3 expression significantly decreased in primary carcinoma and metastatic disease compared with normal and premalignant tissue, suggesting that loss of Gal-3 expression may be associated with the evolution of the disease [52]. Additionally, analyses of the Gal-1 expression in 148 human primary PCa samples by IHC revealed that this Gal was not detected in normal, PIN, or carcinoma cells but accumulated in the stroma, including associated fibroblasts. Gal-1 was significantly increased in the tumor-associated stroma compared with the nonneoplastic gland-associated stroma in more than 21% of the cases. The authors hypothesized that the accumulation of Gal-1 in the stroma of malignant tissue may indicate both a possible role for this Gal in the acquisition of an invasive phenotype and poor prognosis [53].

 Furthermore, alteration in the nuclear/cytoplasmic expression ratio of Gal-3 correlates with PCa progression [54], and decreasing expression of Gal-3 in benign, adjacent-benign, and tumor tissues suggests that Gal-3 expression could be useful for predicting biochemical recurrence [55]. Moreover, Raz's group was the first to show Gal-3 as a cell adhesion molecule involved in tumor progression [29]. In this study, IHC analysis revealed that Gal-3 is cleaved during the progression of PCa, implicating this Gal both as a diagnostic marker and therapeutic target for future disease treatments [29]. Not only Gal-3 expression levels but also its cleavage by matrix metalloprotease (MMP)-2/-9 are related to both breast and prostate cancers and are responsible for tumor growth, angiogenesis, and apoptosis resistance in mouse models [48]. Increased chemotaxis, invasion, and interactions with endothelial cells resulting in angiogenesis and morphologic changes are induced by transfecting BT-459, a Gal-3 negative breast cancer cell line, with either cleavable full-length Gal-3 or its fragmented peptides [48]. Additionally, amino acids 1–62 and 33–250 from cleaved Gal-3 were identified to stimulate migration and morphogenesis of endothelial cells. Thus, cleavage of the aminoterminus of Gal-3 followed by its release in the tumor microenvironment leads in part to breast cancer angiogenesis and progression [48]. In PCa, Gal-3 functions are dependent on both its localization [30] and posttranslational modifications such as cleavage and phosphorylation. Gal-3 can be phosphorylated at Tyr-107 by c-Abl and then cleaved between Tyr107 and Gly108 by prostate-specific antigen (PSA) [49]. Similarities in the role of cleaved Gal-3 with breast cancer could be postulated: Gal-3 cleavage results in loss of lectin multivalency while preserving its carbohydrate binding activity. The authors also showed that Tyr-107 phosphorylation by c-Abl affects Gal-3 cleavage by PSA and influences the localization and role of this lectin in PCa [49]. 

An evaluation of Gal-3 expression in tissue microarrays prepared from 83 tumor, 78 adjacent-benign, and 75 benign tissues obtained from 83 patients who had undergone prostatectomy for clinically localized PCa suggests that the expression of this lectin could be used as predictor of biochemical recurrence [55]. In this study, multivariate analysis (including age, Gleason score, T stage, seminal vesicle invasion, or pre-operative PSA and Gal-3 staining) demonstrated nuclear and cytoplasmic localization of Gal-3 in benign, adjacent-benign and tumor tissues with a significant decrease of its expression from benign to adjacent-benign, and to tumor tissues. These results convincingly demonstrate that Gal-3 staining intensity correlates with biochemical recurrence.

As Gal-1 and Gal-3 are the two best studied Gal members in cancer and particularly in PCa, we wondered if other Gals could show a particular profile of expression. As Gals play fundamental, although divergent, roles in diverse tumor microenvironments [57], identification of the “galectin-specific signature” of PCa is critical for diagnostic, prognostic, and therapeutic purposes. For this, we decided to study the expression of almost all Gals in human prostate cancer cells. Firstly, in order to delineate the Gal expression profile of PCa progression, we examined the Gal transcriptional pattern of several human PCa cell lines, which are representative of different stages of the disease. These include LNCaP and castration-resistant cell lines 22Rv1 or PC-3, which either express or do not express the androgen receptor (AR), respectively, and display a more aggressive behavior *in vivo *[58–60]. Total RNA was extracted in the log phase of growth in culture and analyzed by quantitative RT-PCR. Remarkably, Gal-1 was found to be the most abundantly expressed Gal in all cells analyzed and its expression was higher in those PCa cells exhibiting a more aggressive behavior *in vivo. *Gal-8 mRNA, which has been postulated as a PCa marker [42, 61], was found to be ubiquitously expressed, although being at modest levels in all cell lines tested. Gal-3 mRNA was only detected in androgen-independent, AR negative PC-3 cells. Transcripts for all other Gal family members (Gal-2, -4, -7, -9, -10, and 13), however, were found to be expressed at very low levels. To further delineate “the galectin-specific PCa signature,” we profiled expression of Gals at the protein level (mainly focusing on those members of the family showing higher transcriptional levels). Immunocytochemical analysis of PCa cells confirmed that Gal-1 is the most abundantly expressed Gal in all PCa cells analyzed showing a pronounced upregulation in stages of more aggressive behavior. On the other hand, Gal-3 was predominantly expressed in the PC-3 cell line, and Gals-8 and -9 showed the modest expression in all cell lines analyzed. In agreement with transcriptional profiles, other Gal family members showed lower levels of protein expression. Altogether, these results indicate a fine regulation of Gal expression, mostly at the transcriptional level, in PCa cells characterized by distinct phenotypes, hormone-dependence, and aggressive behavior [23].

**Figure 4:**
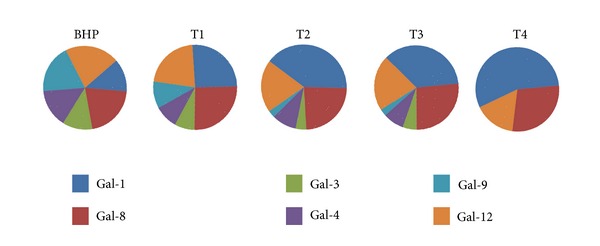
Profile of expression of galectins through PCa evolution. Radical prostatectomies were classified according to TNM scale. Specimens (*n* = 61) covered all stages of prostate cancer evolution, including T1 (tumor detected in less or 5% of the tissue), T2 (tumor confined to the prostate), T3 (tumor that extends beyond the prostatic capsule), and T4 (tumor that invades structures other than seminal vesicles), in addition to BHP. Immunohistochemistry was conducted on paraffin-embedded tissue samples as previously described [23]. The figure shows proportional expression of each Gal at different stages of PCa.

The differential expression of Gals in PCa cell lines prompted us to investigate the profile of these lectins in biopsies obtained from 60 patients naive of any therapeutic treatment. Samples were classified according to TNM classification (UICC, 2002). A large spectrum of PCa phases, T1, T2, T3, and T4 in addition to a benign stage (BHP), were represented. Gal expression was analyzed by IHC in paraffin embedded tissue samples. These results are summarized in Figure 4 and show the evolution of the expression of Gals, essentially Gals-1, -3, -4, -8, -9, and -12 during the progression of PCa. Similar to tumor cell lines, Gal-1 exhibited the highest expression levels which increased progressively during tumor evolution towards more aggressive stages. Further analysis revealed that, in addition to its expression in tumor cells, Gal-1 is also expressed, although at a lower levels, in tumor-associated stroma and normal adjacent tissue. These findings broaden the results reported previously by Ellerhorst et al. [52] and Clausse et al. [24], who showed selective Gal-1 expression in endothelial cells (EC) in PCa. On the other hand, although typically expressed at lower levels, Gals-3, -4, -9, and -12 gradually decrease as the disease advances. Conversely, Gal-8 was highly expressed, but no apparent regulation could be observed during disease progression. Thus, a “galectin-specific signature” characterized by up-, down-, or nonregulated family members delineates PCa progression in patients biopsies, suggesting novel biomarkers of disease evolution. Particularly, we show for the first time Gal-1 expression as a hallmark of PCa aggressiveness, suggesting a major target for anticancer therapies [23]. These changes on Gal profiles may have important impacts on tumor biology as they are reported to affect several cellular processes, developed in the following chapters.

## 4. Effects of Galectins on Apoptosis

Galectins have contrasting effects on apoptosis. While Gal-1 is an apoptosis promoter, Gal-3 shows both pro- or antiapoptotic effects depending on its subcellular localization in PCa. For instance, induction of differentiation and apoptosis by butyrate was investigated in four human PCa cell lines including LNCaP [21]. Treatment of PCa cells with butyrate resulted in increased Gal-1 expression in a time- and dose-dependent manner followed by induction of apoptosis. As LNCaP cells do not express (or express low levels of) Gal-1, transfection with a Gal-1 expression vector inhibits LNCaP cell growth and increases the apoptosis rate. Therefore, Gal-1 may function downstream in the pathway of butyrate-induced differentiation and apoptosis [21].

It is well demonstrated that Gal-1 could act extracellularly to induce apoptosis in glycopermissive cells [62]. In PCa, Gal-1-induced apoptosis is highly dependent on the O-glycosylation of cells. Expression of alpha-2,3-sialyltransferase-1 blocks O-glycans elongation and protects LNCaP subclone from Gal-1-induced apoptosis [22]. This original work shows that the expression of Gal *per se* is not the final determinant of cell apoptosis; instead, regulation of lectin expression and the glycan repertoire determines the final phenotype, sensitivity, or resistance to apoptosis induction. 

In an opposite way, Gal-3 has a dual role in controlling cell apoptosis depending on the PCa cell model. To study whether Gal-3 regulates drug-induced apoptosis, Raz's group either transfected LNCaP cells with Gal-3 or silenced Gal-3 expression in PC-3 cells. They tested sensitivity to cis-diammine-dichloroplatinum and showed apoptosis induction in Gal-3 expressing LNCaP, more precisely through inhibition of cytochrome c release and caspase-3 activation [28]. On the contrary, Gal-3 knockdown in PC-3 cells leads to cell-cycle arrest at G(1) phase, upregulation of nuclear p21, and hypophosphorylation of the retinoblastoma tumor suppressor protein (pRb), with no effect on cyclin D1, cyclin E, cyclin-dependent kinases (CDK2 and CDK4), and p27 protein expression levels [29].

More interestingly, Gal-3 also shows a dual role upon its subcellular localization promoting or alternatively inhibiting apoptosis in Gal-3 transfected LNCaP cells. While nuclear Gal-3 targeting allowed by fusion with nuclear localization sequences was found to have proapoptotic properties, the cytoplasmic form is antiapoptotic and promotes tumor progression [30]. These results clearly demonstrate a fine participation of Gals in the control of survival and proliferation processes in tumor cells.

## 5. Galectins in Cancer Cell Adhesion and Metastasis

During tumor progression, malignant cells acquire the ability to overcome cell-cell adhesion and invade surrounding tissues, a state known as metastatic disease. It is important to understand this process and identify inducers of tumor metastasis in order to develop treatments that target metastatic cells for long-term tumor regression.

Galectin-1 is involved in numerous biological functions including capillaries formation. Gal-1 is expressed by endothelial cells (EC) from capillaries infiltrating the tumor tissue in 64% of the cases of 100 human prostate carcinoma samples, but in only few cases (7%) in endothelial cells in the adjacent nontumoral stroma. These results strongly suggest that tumor cells induce Gal-1-expressing EC allowing tumor adhesion to vessel endothelium. To verify this hypothesis, Clausse and colleagues incubated HUVECs cells with conditioned media from PC-3 or DU145 prostate carcinoma cells and they observed a significant increase of Gal-1 protein expression. Additionally, both PC-3 conditioned medium and recombinant Gal-1 induced increased PC-3 cells adhesion to EC, while conditioned media complemented by an anti-Gal-1 antiserum abolished this modulation [24]. It is important to note that no cell adhesion to EC was observed when normal lymphocytes were used instead of PC-3 cells under the same conditions. This places Gal-1 as a molecular link of the specific EC/tumor interaction and suggests an additional tumor-based immune escape mechanism. In the same way, expression of Gal-1 in the cell surface of LNCaP also showed the lectin ability to modulate adhesion to the ECM [25] revealing a general function of Gal-1 in PCa cell-matrix interactions.

Capillary formation is essential for tumors to obtain nutriments as well as for migration and the metastasis process. Interactions of metastatic cancer cells with blood vessels are critical during early stages of cancer metastasis but also in the lodge of tumor cells in specific organs and tissues. In 2005, Glinskii and colleagues demonstrated that mechanical entrapment alone, in the absence of tumor cell adhesion to blood vessel walls, is not sufficient for metastatic cell arrest in the microvasculature of the target organ. The analysis of the frequency and location of fluorescent tumor cells in different organs and tissues following intravenous inoculation revealed that PCa cells go into a wide variety of tissues and organs but not to the lung capillary bed. Results showed that arrest of metastatic cells in target organ microvessels is not a consequence of mechanical trapping, but is supported predominantly by intercellular adhesive interactions mediated by cancer-associated Thomsen-Friedenreich (TF) antigen and Gal-3 [35]. Additionally, carbohydrate moieties of the TF antigen (Gal*β*1,3GalNAc) on the surface of endothelial cells could be efficiently recognized by Gal-3, thus priming them for harboring metastatic cancer cells [34].

In mCRPC, bones are a privilege site for the metastatic disease that causes osteoblastic growth. However, the mechanisms that contribute to bone metastasis are poorly understood. It was suggested that the bone provides a favorable environment for PCa cells growth and that tumor cells preferentially bind to bone marrow EC. To verify this hypothesis, cancer cell adhesion to a human bone marrow endothelial (HBME-1) cell and EC lines from other organs was assessed. *In vitro*, PCa cells adhered preferentially to HBME-1 when compared with endothelium derived from other sources, and this adhesion was inhibited by anti-Gal-3 and anti-LFA-1 sera [36]. These data showed that bone metastasis of PCa cells is essentially caused by their preferential binding to bone marrow endothelial cell and in part mediated by cell-cell adhesion via Gal-3 [36]. As in non-small-cell lung cancer, collagen XXIII is a transmembrane protein previously shown to be upregulated, at least in part, through Gal-3, and whose expression facilitates metastases formation in PCa model [37]. This suggests a potential role for collagen XXIII in combination with Gal-3 in mediating metastasis by facilitating cell-cell and cell-matrix adhesion as well as anchorage-independent cell growth.

Being currently incurable, PCa metastasis has a remarkable ability to spread to the skeleton. Advanced PCa cells are essentially characterized by bone metastasis that predominantly causes an osteolytic phenotype. In a PC-3 cellular model, it has been shown that both the conditioned media from these PCa cells containing Gal-1 and recombinant Gal-1 inhibited the osteoblastic proliferation and differentiation of human bone marrow stromal (hBMS) cells. Thus, the authors hypothesized that Gal-1 could be involved in the osteoblastic response caused by PCa cells metastasizing to the bone, by affecting the matrix mineralization [26]. To date, studies in animal models still fail to demonstrate the role of Gal-1 in PCa metastasis process. 

Gal-3 is the only member of this family of lectins that was studied *in vivo*: Gal-3 expression drives spontaneous metastasis using rat PCa models such as Dunning or Copenhagen rat [38]. The oral administration of modified citrus pectin (MCP, pH-modified), a soluble component of plant fiber derived from citrus fruit, revealed inhibition of cell-cell interactions mediated by cell surface carbohydrate-binding Gal-3 molecules. In fact, the presence of Gal-3 in Dunning PCa cell lines (MAT-LyLu cells) and primary human prostate carcinoma was demonstrated by immunoblotting and IHC. Lung metastatic colonies were observed after subcutaneous injections of MAT-LyLu cells in posterior legs of male Copenhagen rats, while continuous administration of MCP in drinking water reduced the number of lung metastases. MCP had no effect on the growth of the primary tumors suggesting that the reduction of lung metastases was caused by both interference with migration or tumor adhesion such as cell adhesion to EC, and the spreading of tumor cells. As MCP is not an exclusive inhibitor of Gal-3, further studies are still required to specifically target this galectin and determine its role in normal and cancerous prostate tissues and the ability of Gal-3 targeting to inhibit prostate metastasis in animal models. As we previously described, interactions mediated by the cancer-associated TF glycoantigen and Gal-3 play an important role in several rate-limiting steps of cancer metastasis such as cell adhesion to bone marrow endothelium, homotypic tumor cell aggregation, and clonogenic survival and growth [34, 35], and it was only recently shown that Gal-3 influences bone metastasis in a mouse model after intracardiac injection of luciferase-expressing PC-3 cells in nude mice [39]. Indirect targeting of Gal-3 by using daily intraperitoneal administration of Lac-l-Leu, which binds and inhibits Gals by mimicking essential structural features of the TF-Ag, affects PCa cell adhesion to bone marrow endothelium, homotypic aggregation, transendothelial migration, clonogenic growth, and final spreading of tumor cells to the skeleton [39]. These results were recently confirmed by others demonstrating inhibition of tumor-endothelial cell interactions and lung metastasis using TFD100 (a purified glycopeptide acting as competitor in Gal-3 binding to TF-Ag on the surface of most cancer cells [33]), or using MCP in combination with other drugs such as ProstaCaid [41]. Altogether, they highlight the impact of Gal-3 on invasive behavior in human PCa cells *in vitro*. As Gal-3 is not expressed in advanced stages of the disease, other factors act to promote spreading of tumor cells and have to be identified to attempt to cure mCRPC patients.

Galectin-8 was initially called Prostate Cancer Tumor Antigen-1 (PCTA-1) because of its exclusive expression in neoplastic prostate cells and its absence in normal prostate tissue. In fact, Gal-8 levels of expression positively correlate with certain human neoplasms [42]. Gal-8, like other galectins, is a regulator of cell adhesion depending on its formulation. Thus, immobilized protein acts as a potent matrix protein in promoting cell adhesion by ligation and clustering of a selective subset of cell surface integrin receptors and triggering signaling cascades including Tyr phosphorylation of focal adhesion kinase and paxillin [63]. In contrast, when present in excess as a soluble ligand, Gal-8 forms complexes with integrin that negatively regulates cell adhesion and tumor properties such as growth and metastasis [43]. No current evidence exists about these potential roles of Gal-8 in animal models to understand why Gal-8 is only expressed in prostate tissue at neoplastic stages.

Interestingly, glycosyltransferase-mediated regulation of carbohydrate expression on cell membrane-glycoconjugates has been recently shown to be involved in migration and invasion properties of the PC-3 cell line. It is well known that Gals link to tri- and tetrabranched N-glycans forming multivalent lattices that enhance cell surface residency of growth factor receptors and focal adhesion turnover. Silencing N-acetylglucosaminyltransferase I (MGAT1, the first enzyme of N-glycans biogenesis) by RNA interference in PC-3 was enough to inhibit cell invasion by affection of focal adhesion and microfilament organization, thus generating a less motile phenotype. More importantly, orthotopic injection of MGAT1-silenced PC-3 in nude mice revealed a decrease in primary tumor growth and poor incidence of lung metastases as well [64]. Not only N-glycans should be considered as potential regulators of Gal functions in PCa but also O-glycosylation confers LNCaP cells susceptibility to Gal-1-induced apoptosis [22].

## 6. Galectins as Inducers of Tumor Angiogenesis

Cancer metastasis involves a series of steps including angiogenesis, detachment of tumor cells from the primary tumor, intravasation, evasion of host defense, arrest and attachment at a distant site, extravasation, dormant survival, and establishment of new growth. During extravasation, tumor cells bind to endothelial cells through protein, carbohydrate interactions and penetrate through the endothelium and basement membrane. Besides providing tumors with nutrients, newly formed capillaries constitute a potential escape route for tumor cells, thus favoring metastatic dissemination, and also provide an access to host immune cells.

Analysis of Gal-1 expression in EC from 100 PCa patients who had undergone a radical prostatectomy for localized prostate cancer (Gleason score from 2 to 10) revealed increased frequency of Gal-1 expression in capillaries infiltrating the tumor compared to those present in the non tumoral adjacent tissue [24]. Although EC do express Gal-1, *in vitro* culture of HUVEC cells in normal medium complemented by conditional media from PC-3 or DU145 led to enhanced Gal-1 expression in EC [24]. These results demonstrate that secreted factors from tumor cells influence Gal-1 expression in capillaries cells and promote specific attachment of tumor cells to EC. This heterotypic cell interaction is essentially due to Gal-1 produced by tumor cells as Gal-1 blocking antibodies inhibited this effect [24]. However, further experiments are required to unveil the role of this interaction in the evolution of the disease.

Because Gal-1 expression is associated with PCa aggressiveness and has emerged as a novel proangiogenic factor in other tumor types [65, 66], we decided to further examine whether expression of this lectin correlates with the frequency of blood vessels in low grade or high grade human PCa [23]. For this purpose, we evaluated coexpression of Gal-1 and CD34 by IHC analysis of a human PCa tissue array comprised of 29 paired cores of invasive PCa. A positive correlation between Gal-1 and CD34 was selectively detected in arrays of human PCa, but not in arrays of human breast cancer which served as control, suggesting a tissue-specific proangiogenic effect of this lectin in cancer. This selectivity is consistent with the ability of Gal-1 to induce angiogenesis of oligodendroglioma [65], B16 melanoma [67], and Kaposi's sarcoma [68], but not Lewis lung carcinoma [69]. This correlation between Gal-1 expression and the number of blood vessels was also verified when the tumor compartment was compared to nonmalignant areas and was even more pronounced in high grade compared to low grade tumors [23]. Complementary to what was previously reported by Clausse and colleagues [24], we demonstrated that tumor cells are the major source of Gal-1. While some inconsistencies are observed between studies addressing the relative expression of Gal-1 by stroma versus tumor cells probably by differences in methodological approaches, its functional impact on other cancers such as melanoma or lung carcinoma was elegantly assessed in mice by comparing the functionality of these cellular compartments under Gal-1 deficiency or wildtype conditions. Those results clearly demonstrated tumor as the main Gal-1 source in controlling tumor growth [69]. Another possibility that must be taken into consideration is the hypothesis that EC are able to capt tumor derived-Gal-1 through mechanisms that must be fully understood [67].

Given the promising therapeutic value of anti-angiogenic strategies in advanced androgen-refractory PCa [70], we were prompted to examine the role of Gal-1 in PCa angiogenesis. We first evaluated the effect of conditioned medium obtained from 22Rv1 (PCa CM), a Gal-1-positive PCa cell line, on* in vitro* tubulogenesis. PCa CM induced the formation of tubular-like structures when added to EC. The involvement of Gal-1 in this process was assessed by using an anti-Gal-1 neutralizing mAb, which considerably reduced the formation of these structures [23]. These *in vitro *effects of Gal-1-expressing PCa cells on endothelial cell morphogenesis prompted us to investigate the role of this lectin in angiogenesis* in vivo*. Our experimental approach consisted in the s.c. injection of 22Rv1 PCa cells in Matrigel plugs. Importantly, we were able to differentiate the source of Gal-1 (tumor and microenvironment versus tumor alone): firstly, a blocking anti-Gal-1 mAb was added to the mix (total Gal-1 inhibition independently of its source is to be expected); alternatively, we used 22Rv1 tumor cells transduced with a human specific Gal-1 shRNA-coding lentivirus (thus inhibiting tumoral Gal-1 expression alone). A marked reduction of microvessel density was observed using both experimental approaches, indicating that tumor cells are the main source of Gal-1, at least at early time points of tumor implantation and neovascularization. Confirming this reasoning, intermediate effects were observed when Gal-1 was partially downregulated in PCa cells. Altogether, these *in vitro* and *in vivo* results reveal a key role of Gal-1 in PCa-induced angiogenesis. More importantly, we showed that *in vivo *silencing of Gal-1 expression by tumor cells does not interfere with other pro- or anti-angiogenic factors such as VEGF or thrombospondin and bFGF, revealing the preponderant role of Gal-1 in promoting PCa neovascularization and suggesting Gal-1 as a new potent target for clinic therapeutic approaches in advanced PCa patients [23].

Gal-3 could also act as an angiogenic inducer by recognizing the TF disaccharide antigen present on the surface of most cancer cells. Using a purified glycopeptide TFD100 that binds Gal-3 with picomolar affinity, the authors blocked Gal-3-mediated interactions and inhibited angiogenesis of PC-3 tumors in mice [33]. In this PC-3 model, Gal-4 and Gal-9 also efficiently bind to TFD100 and thus are implicated in PCa. Consequently, silencing of these molecules causes strong reduction of *in vitro* tubulogenesis and VEGF-induced blood vessels formation in Matrigel plug assays [33]. Altogether, these results highlight a major role of the interactions between Gals and their corresponding glyco-ligands in determining tumor-associated angiogenesis.

## 7. Galectins as Immune Tolerance Inducers in Prostate Cancer 

An efficient immune response against pathogens or tumors needs effective egress of lymphocytes from the blood into the target tissue. This process is allowed in part by specific EC proteins promoting lymphocyte adhesion to and migration across endothelium. Other molecules negatively regulate transendothelial migration of lymphocytes. Gal-1 is one of the best studied members that acts as inducer of immune tolerance in cancer [10]. As it was previously showed, Gal-1 expression could be induced in EC by neighbor tumor cells [24], but it is well known that this lectin could regulate the inflammatory setting, modulating T cell cytokine production and triggering T-cell death [17, 27]. Migration of T-cell lines through Matrigel is inhibited by EC treated with either PCa conditioned media containing Gal-1 or with Matrigel coated with recombinant Gal-1. This inhibition is reverted by using an anti-Gal-1 serum, demonstrating that transendothelial migration of T cells is negatively regulated by Gal-1-producing EC. More importantly, the inhibition is due to decreased adhesion of T-cells to Gal-1 expressing EC rather than T cell death. In fact, T-cell treatment with benzyl-a-GalNAc, which reduces core 2 O-glycan expression thus blocking Gal-1 recognition, inhibits Gal-1-induced T cell death. Polarization of CD43 molecule on T cells is essential for T cell migration. Interestingly, Gal-1-coated ECM enhanced clustering of CD43, which contributes to the inhibitory effect on T-cell migration [27]. The role of Gals as active controllers of immunological tolerance in PCa is a field that is still open to new discoveries. 

## 8. Galectins as Molecules with Prognosis and Therapeutic Value in Prostate Cancer: From Animal Models to Clinical Settings

PCa is curable only when detected in its early stages as a result of both prostate-specific antigen (PSA) blood test screening and digital rectal exam. When patients suffer from metastatic disease and undergo mCRPC, docetaxel-based combination chemotherapy is the only available therapy that has demonstrated a survival benefit in 50% of these advanced stages of PCa. Moreover, FDA approved new therapies, most are being evaluated on clinical trials and are based on targeting the angiogenesis, the tumor microenvironment, or immunotherapy [71]. All these drugs showed improved overall survival (OS) of the mCRPC patients for 3–5 months. One of these drugs is PROVENGE (sipuleucel-T), a dendritic cell-based immunotherapy targeting the prostatic acid phosphate (PAP), an antigen expressed in more than 95% of PCa [72]. The treatment consists in *ex-vivo* loading of autologous dendritic cells with the recombinant antigen which is PAP-GM-CSF: chimera of PAP and the granulocyte-macrophage colony-stimulating factor (GM-CSF), an immune adjuvant. This new treatment is only intended for men with asymptomatic or minimally symptomatic and metastasized PCa that are resistant to standard hormone treatment. In controlled and multicenter clinical trials, several adverse events have been reported in the PROVENGE group, which include acute infusion reactions (occurring within 1 day of infusion) and cerebrovascular events. The most common adverse events (incidence ≥ 15%) reported were chills, fatigue, fever, back pain, nausea, joint ache, and headache [73].

Xtandi (enzalutamide), an androgen receptor antagonist, is a new PCa treatment approved by FDA in August 2012 to treat mCRPC patients, with spreading or recurred cancer even with medical or surgical therapy and resistant to docetaxel-based chemotherapy.

Finally, Xofigo was approved by FDA in mid-2013 for patients with CRPC, symptomatic metastases that spread to bones but not to other organs as in visceral metastatic disease. Xofigo binds with minerals in the bone to deliver radiation directly to bone tumors, limiting the damage to the surrounding normal tissues. Xofigo is an alpha particle-emitting radioactive therapeutic agent (radium-223 dichloride). The most common side effects were nausea, diarrhea, vomiting, swelling of the leg, ankle, or foot, and blood cells abnormalities with less than 5 months of improved OS. 

Therefore, it is critical to identify new molecular targets to efficiently cure advanced cancers. A limited number of studies consider potential implications of individual Gals in the modulation of the metastatic process, yet the role of these essential proteins *in vivo *is still unclear. Under this scenario, a more deeply comprehension of the influence of Gals in evolution of PCa could help to define new drugs that treat advanced and mCRPC patients. For instance, the ratio of phosphorylated/dephosphorylated Gal-3 might be used as a complementary value to that of PSA for prognosis of PCa [49]. 

From a therapeutic point of view, Gal-3 has been conjugated to the chemotherapy drug 5-Fluoracil and delivered to PC-3 tumors by using a copolymer system named Gal-3-targeted HPMA copolymer-(G3-C12-)5-Fluorouracil conjugates. This drug showed *in vitro* inhibition of PC-3 cell migration after wounding and displayed a potent antitumor activity against PC-3 tumor xenografts in *nude* mice [40]. 

Drug resistance is a major obstacle for PCa therapy, but its underlying mechanisms are not clear, especially in patients with advanced stages of the disease. A comparative proteomic profiling of camptothecin- (CPT-) resistant PC-3 and CPT-sensitive LNCaP human PCa cell lines identified a signature of 144 proteins with different expression levels between the two cell lines that are suggested to contribute to the development of drug resistance [74]. In this respect, Gal-3 is highly expressed in PC-3 cells, whereas it is not detectable in LNCaP. The expression level of these proteins and/or mRNAs could be a useful parameter to evaluate chemotherapy resistance in clinical specimens of PCa [74]. The same conclusion was drawn when Gal-3 silencing induced increased cisplatin-induced apoptosis of PC-3 cells [31]. Resistance to apoptosis is a critical feature of neoplastic cells; Gal-3 either inhibits anticancer drug-induced apoptosis or promotes cell death depending on its subcellular localization. These findings suggest that Gal-3 targeting could improve the efficacy of anticancer drug chemotherapy in PCa [28]. In fact, Gal-3 presents a domain like NWGR anti-death motif of Bcl-2 family which confers antiapoptotic properties through regulation of Bad protein and suppression of the mitochondrial apoptosis pathway [75]. Thus, Gal-3 shows multifunctional oncogenic functions such as the regulation of tumor proliferation, angiogenesis and apoptosis. In this sense, sensibility to proapoptotic agents like cis-platin and etoposide is higher in Gal-3 negative PCa cell lines than in Gal-3 expressing cells [28, 32]. These observations imply that Gal-3 inhibits anticancer drug-induced apoptosis and, consequently, Gal-3 targeting could improve the efficacy of anticancer drug chemotherapy in PCa. However, as Gal-3 is only expressed in early but not in late stages, it is unlikely that this kind of treatment could serve as new curative options for mCRPC patients. 

Recently, we confirmed pioneer studies of Gal-1 as a tumoral marker of poor prognosis for PCa patients [53], and we showed a regulated expression of several Gals with diagnostic value. In fact, strong increase of Gal-1, decrease of Gals-4, -9, and -12, and gradual decrease to complete extinction of Gal-3 expressions could define the stage of PCa progression using IHC as a simple method to analyse Gal expression in available patients samples (Figure 4 and [23]). As shown in Figure 4, comparison of Gal expression could define PCa patient stage: T1, T2-T3, and T4; however, while T1 and T4 could be easily identified, it appears difficult to differentiate T2 to T3 stages since Gal profile is similar between these two intermediate stages.

Galectins are not only shown to be involved in antiapoptotic functions and edition of immune tolerance; several studies clearly revealed their proangiogenic functions in cancers [68, 76–78]. Interactions of metastatic cancer cells with vascular endothelium are critical during early stages of cancer metastasis. Various investigations, not developed in this review due to space limitations, showed Gals as angiogenic-regulatory proteins. For instance, Gal-3 was suggested as a new target to treat breast cancer patients [34], and Gal-8 as a new modulator of EC migration and angiogenesis [79]. In the case of PCa, we recently demonstrated that Gal-1 is the principal inducer of neoangiogenesis [23] and could be used as a novel target for anti-angiogenic therapies in human advanced PCa.

## 9. Conclusion

While localized PCa can be cured, metastatic and advanced prostate cancers pose a significant therapeutic challenge. We and others identified a “galectin-regulated signature” as new prognostic markers and molecular targets of novel therapeutic avenues for preventing metastasis. Tumor metastasis is a multistep process involving several cellular and molecular interactions. Recognition of glycoconjugates by galectins regulates tumor behavior through both intrinsic as well as extrinsic signals involving modulation of homotypic cell aggregation, tumor cell apoptosis, angiogenesis, and tumor immune escape [9]. In fact, Gal-1 expression regulates prostate tumor cell resistance to apoptosis before becoming castration resistant [22]. As shown in breast cancer, Gal-3 containing NWGR amino acid domain sequence as bcl-2 gene family also could act as antiapoptotic protein independently of Bcl-2, Bcl-X_L_, or Bax proteins [80]. Thus, both Gals may contribute to tumor survival and to the selection process that is characteristic of the evolution of this type of cancer. However, not only the tumor itself but also the surrounding tissues and the complex network of stromal, endothelial, and immune cells that interact within the tumor microenvironment should be considered. In this respect, cancer cells not only control the expression of their own Gals but also modulate Gal expression in environmental tissues [24]. Gals regulate cell-cell and cell-ECM interactions. Surprisingly, different members of the family elicit particular and sometimes antagonic effects. In fact, anti-Gal-3 monoclonal antibodies (mAbs) prevent the adhesion of prostate tumor cells to bone marrow endothelial cells [36]. In addition, Gal-1 increases, while Gal-8 reduces tumor cell-ECM interactions [52, 63, 81]. Altogether, these effects may contribute to shaping a favorable tumor microenvironment that allows distant dissemination of transformed cells.

Despite these observations, there are relatively few *in vivo* studies addressing the source and function of Gals and exploring these phenotypes in PCa [35, 38, 82, 83]. In fact, expression levels of Gals-1 and -3 were reported to be associated with the growth and metastatic properties of prostate tumors and may correlate with a poor prognosis [24, 35, 38, 52, 82, 84]. During the last two decades, Gal-3 was the only member of the family whose role has been addressed using *in vivo* experimental models. A key role of this glycan-binding protein in the formation of spontaneous metastasis was demonstrated using peptide inhibitors in experimental animal models of PCa [38, 39]. Moreover, tumor cell expression of Gal-3 has been shown to delineate the transition from benign prostate glands to hormone-resistant malignant disease [84], and its regulated expression is associated with promoter methylation [85]. Silencing Gal-3 results in decreased migration, invasion, and proliferation of PCa cells [29]. Taking into consideration these results, Gal-3 emerges as a key lectin that plays an essential role in the formation of metastases but not in the progression of advanced disease. Moreover, Gals-1 and -3 were both found in the nucleus of cells [86, 87]. The importance of both Gals in presplicing RNA activity has been demonstrated by depleting these lectins from nuclear extracts, which causes inhibition of the splicing activity [88, 89]. These properties and the relation with other nuclear factors such as splicing or transcriptional factors should be explored in PCa to identify new intracellular partners that could represent new therapeutic targets and signaling pathways as well. 

Galectin profile in cancer may be intrinsically determined and consequently considered as biomarkers of cell transformation. In this respect, we can assume that each type of tumor can be associated with characteristic profiles of Gals. Our study on PCa cell lines and patient samples proposes a particular profile of Gals through disease progression [23, 76]. However, tumor cell must be considered in association with its microenvironment. In this respect, most of the cancers are characterized by particular inflammatory processes where bidirectional dialogues between tumor, resident, and infiltrating cells result in changes on cell surface expression of glycans and lectins. It is possible that Gals may be an acute phase reactants produced in response to tumor-associated stress. Under this view, changes in Gal profiles could represent an epiphenomenon due to associations with inflammation. Whatever the real cause/effect, changes in Gal expression may be exploited as accurate tools for diagnosis, prognosis, and probably therapeutic purposes in cancer.

In spite of considerable progress in dissecting the functions of individual Gals, an integrated portrait of the “galectin signature” of the human PCa microenvironment is still missing. In addition, it is not clear which cellular compartment expresses a given Gal and when this expression is required for cancer progression. While only Gals-1 and -3 were extensively studied in PCa, these Gals have been already shown to be potential new factors that participate directly in PCa progression and cancer drug resistance. In fact, Raz's group showed that phosphorylated Gal-3 is responsible for drug resistance in PCa and should be considered as new target to improve efficiency of chemotherapeutic agents such as cisplatin and etoposide [32]. We demonstrated that Gal-1 expressed by the tumor is essential and sufficient to promote neovascularization independently of classical angiogenic pathways. This strongly supports the idea of Gal-1 targeting as a new anti-angiogenic therapy for advanced PCa. Additionally, we showed that Gals-4 and -12 suffered decreased expression through PCa evolution, but the role of these Gals in PCa is a field that lacks a more deeply understanding. 

Finally, Gal-8 is another tandem-repeat Gal of importance in PCa. This Gal has been defined first in 1996 [42] and 2000 [61] as PCa biomarkers and six years after as a potential responsible for hereditary PCa [90]. To date, no study has been performed to understand why this particular Gal is generally expressed in all tissues but it only turned on in neoplasic prostatic tissues and is absent in normal prostate. Despite these important characteristics, studies about the role of Gal-8 in PCa are still missing. Thus, its use as a therapeutic target in this disease should be further explored. 

## Figures and Tables

**Table 1:** Summary of reported galectin functions in prostate cancers.

Galectin	Tumor cell growth/survival/apoptosis		Metastasis		Immune response	
	*In vitro *	*In vivo *	*In vitro *	*In vivo *	*In vitro *	*In vivo *
Gal-1	Promotes apoptosis in LNCaP [21] O-glycosylation protects PCa cell from Gal-1-induced apoptosis [22] Promotes tubulogenesis [23]	Gal-1 principal inducer of neovascularization [23]	Promotes cell adhesion to ECM, EC [24, 25] Osteoblasts proliferation and differentiation, effects inhibited by IGF [26]	?	Invasion of T cell in matrigel assays and adhesion of T cell to Gal-1-expressing EC [27]	?

Gal-3	Promotes apoptosis or survival depending of cell subcellular localization or cell type [28–30] Drug resistance [31, 32]	Gal-3 as inducer of angiogenesis [33]	Interaction with blood vessel allowing metastasis process such as arrest in certain organs [34, 35] PCa cell with preferential binding to HBME through collagen XXIII and Gal-3 could explain bone metastasis [36, 37]	Anti-Gal-3 Abs or MCP inhibits spontaneous metastasis in Copenhagen rat-injected Dunning rat PCa cells [38], influences bone metastasis as indirect inhibition of Gal-3, and inhibits skeleton metastasis after Luc-PC-3 intracardiac injection [39] Using Gal-3 inhibitors inhibits tumor growth or lung metastasis [33, 40, 41]	?	?

Gal-8	Exclusive expression at the neoplasic stage in prostate tissue (PCTA-1); links to integrin to inhibit cell adhesion [42]	?	Links to integrin to inhibit cell adhesion and promote metastasis and cell spreading. In contrary, in soluble form Gal-8 promotes cell-adhesion to ECM [43]	?	?	?

Gal-4	Gal-4 as inducer of tubulogenesis [33]	?	?	?	?	?

Gal-9	Gal-9 as inducer of tubulogenesis [33]	?	?	?	?	?

Gal-12	?	?	?	?	?	?

Others Gals	?	?	?	?	?	?
